# *RBX1* loss sensitizes tubo-ovarian, high-grade serous ovarian cells to CDK2 inhibition by SNS-032

**DOI:** 10.3389/fcell.2026.1781550

**Published:** 2026-04-28

**Authors:** Ally C. Farrell, Lukas A. Lam, Helen Chen, Nicole M. Neudorf, Babu V. Sajesh, Chloe C. Lepage, Zelda Lichtensztejn, Kirk J. McManus

**Affiliations:** 1 Department of Biochemistry and Medical Genetics, University of Manitoba, Winnipeg, MB, Canada; 2 Paul Albrechtsen Research Institute, CancerCare Manitoba, Winnipeg, MB, Canada

**Keywords:** CDK2, RBX1, SNS-032, synthetic lethality, tubo-ovarian high-grade serous carcinoma

## Abstract

Tubo-ovarian, high-grade serous carcinoma (HGSC) is the most lethal gynecological malignancy, with limited targeted therapies and poor outcomes. Heterozygous loss of *RBX1* occurs in approximately 81% of HGSCs and drives chromosome instability and cellular transformation. Here, we identify *CDK2* as a novel synthetic lethal (SL) interactor of *RBX1* in clinically relevant *RBX1* ^+/−^ fallopian tube secretory epithelial cell models. Genetic silencing or pharmacologic inhibition of CDK2 with siRNA duplexes or SNS-032, respectively, selectively reduced viability and induced cytotoxicity in *RBX1* ^+/−^ cells, with significantly lower EC_50_ values compared to controls. Importantly, in two malignant HGSC cell lines (COV362 and OVCAR-3), we further observed that *CDK2* silencing or SNS-032 treatment in combination with *RBX1* silencing induced significant reductions in cell numbers, thereby extending the SL interaction to established HGSC models. Mechanistically, SNS-032 treatment led to increased DNA double-strand breaks and apoptosis, as evidenced by increased numbers of γ-H2AX foci and cleaved Caspase-3 signal intensities. To our knowledge, this is the first demonstration of a SL interaction that exploits a heterozygous disease state in HGSC. These findings highlight CDK2 inhibition as a promising precision medicine strategy for *RBX1*-deficient tumors, broaden the applicability of SL approaches beyond homozygous gene loss, and provide strong preclinical rationale for further therapeutic development.

## 1 Introduction

Tubo-ovarian, high-grade serous carcinoma (HGSC; formerly referred to as high-grade serous ovarian cancer) is the most frequently diagnosed epithelial ovarian cancer. Approximately 70%–80% of patients are initially diagnosed at advanced stages (III or IV), and chemotherapy is typically employed to treat residual disease following cytoreductive surgery (Bowtell, 2010; Cortez et al., 2018). First-line chemotherapeutics, such as carboplatin (platinum-based agent) plus paclitaxel (microtubule poison) are associated with side effects and despite initial responses, >70% of patients will experience tumor recurrence and ultimately succumb with drug resistant disease (Lisio et al., 2019; Morden et al., 2021). Given these statistics, there is an urgent need to identify novel drug targets so that improved precision medicine strategies can be developed to more effectively combat the disease.

Chromosome instability (CIN) is form of genome instability and an enabling hallmark of cancer that is proposed to drive disease pathogenesis (Hanahan and Weinberg, 2011). It is defined as an increase in the rate at which whole chromosomes or large chromosome fragments are gained or lost and is a driver of genetic and cell-to-cell heterogeneity (Geigl et al., 2008; Thompson et al., 2010; Thompson et al., 2020; Bungsy et al., 2021; Lepage et al., 2021; Morden et al., 2021; Palmer et al., 2021). Recently, we determined that 90%–95% of HGSCs exhibit CIN (Penner-Goeke et al., 2017; Morden et al., 2021) and we posit that employing a synthetic lethal (SL) paradigm (reviewed in Sajesh et al., 2013b; O’Neil et al., 2017) to target the molecular determinants of CIN (i.e., the aberrant genes, proteins and pathways) will reduce off target effects and enhance on-target specificity and cancer cell killing. In this regard, SL strategies have recently entered the clinic as breast and ovarian cancers with *BRCA1*/*BRCA2* mutations (or other homology direct repair defects) are now being treated with PARP1 inhibitors, such as Olaparib and Rucaparib.

The SKP1-CUL1-F-box (SCF) complex is a multi-subunit E3 ubiquitin ligase that regulates the degradation of key proteins involved in cell cycle control, DNA damage response, and oncogenic signaling (Yeh et al., 2018; Thompson et al., 2020; Bungsy et al., 2021; Lepage et al., 2021; Palmer et al., 2021; Thompson et al., 2021; Neudorf et al., 2022). The SCF complex is an E3 ubiquitin ligase that polyubiquitinates proteins for degradation via the 26S proteosome (Kurz et al., 2005; Campos Gudino et al., 2021; Palmer et al., 2021). The complex includes three core components (SKP1, CUL1, and RBX1) and one of 69 variable F-box proteins that confer substrate specificity to the complex (Kurz et al., 2005; Campos Gudino et al., 2021; Palmer et al., 2021). Loss or reduced expression of SCF members disrupts protein homeostasis, promotes CIN, and contributes to cellular transformation that is suspected to promote early disease development (Yeh et al., 2018; Thompson et al., 2020; Bungsy et al., 2021; Lepage et al., 2021; Palmer et al., 2021; Thompson et al., 2021; Neudorf et al., 2022). Notably, heterozygous loss of *RBX1*, a core SCF component, is observed in ∼81% of HGSCs and reduced expression following siRNA-based silencing or heterozygous loss through CRISPR/Cas9 editing is associated with increases in Cyclin E1 abundance, CIN, and cellular transformation in fallopian tube secretory epithelial (FT) cells (Campos Gudino et al., 2021), the cellular precursor of HGSC (Karst et al., 2011; Perets et al., 2013; Labidi-Galy et al., 2017).

Building on our prior work demonstrating that *RBX1* haploinsufficiency corresponds with increased Cyclin E1 protein abundance, promotes CIN, and drives cellular transformation (Bungsy et al., 2021), we hypothesized that targeting vulnerabilities specific to *RBX1*-deficient cells could provide a novel therapeutic strategy for HGSC. To this end, we employed a SL paradigm to identify candidate drug targets that selectively target *RBX1* ^+/−^ cells. Through bioinformatic analysis of a SL database and functional screening, we identified cyclin-dependent kinase 2 (*CDK2*) as a novel SL interactor of *RBX1*. CDK2 is a key regulator of the G1/S cell cycle transition, functioning in complex with Cyclin E1, which accumulates following siRNA-based silencing or CRISPR/Cas9-mediated knockout of *RBX1* (Bungsy et al., 2021). We prioritized CDK2 for further study based on its established role in cell cycle progression and its functional connection to Cyclin E1, whose dysregulation is a hallmark of *RBX1*-deficient HGSC. Using two clinically relevant *RBX1* ^+/−^ FT cell models, we demonstrate that genetic silencing of *CDK2* or pharmacologic inhibition with SNS-032 preferentially reduces the number of *RBX1* ^+/−^ cells, with SNS-032 treatments underlying increases in DNA double-strand breaks (DSBs) and apoptosis. Importantly, extending these findings to two malignant HGSC cell lines (COV362 and OVCAR-3), we determined that *CDK2* silencing or SNS-032 treatment in combination with *RBX1* silencing resulted in a significant decrease in cell numbers, supporting the translational relevance of this vulnerability in established malignant settings. To our knowledge, this is the first demonstration of a SL interaction exploiting a heterozygous disease state in HGSC. Together, our results suggest that targeting CDK2 in *RBX1*-deficient tumors may represent a new precision medicine approach and expand the therapeutic utility of SL beyond homozygous gene loss.

## 2 Materials and methods

### 2.1 Bioinformatic analyses of HGSC patient datasets

Publicly available data from serous carcinoma patients were extracted from the Cancer Genome Atlas (TCGA) Pan-Cancer Atlas (Hoadley et al., 2018) dataset using cBioPortal (Cerami et al., 2012; Gao et al., 2013). OncoQuery Language commands were applied to filter for cases with *RBX1* shallow deletions (heterozygous loss of one allele; HETLOSS) and cases with no detected copy number changes or mutations (diploid controls). For each case, clinical and molecular features including *RBX1* mRNA expression levels (derived from a total of 201 patient samples), fraction of genome altered (derived from a total of 398 patient samples), and aneuploidy scores derived from a total of 391 patient samples) were downloaded and imported into Prism v10 (GraphPad), where statistical comparisons were performed and graphs generated. A two-tailed unpaired t-test was applied to compare *RBX1* transcript levels between shallow deletion and diploid cases, while Mann-Whitney tests compared cases with *RBX1* shallow deletions and diploid controls for the fraction of genome altered and aneuploidy score. In all instances, a p-value <0.05 was considered statistically significant (*, p-value < 0.05; **, p-value < 0.01; ***, p-value < 0.001; ****, p-value < 0.0001). Associations between *RBX1* expression and patient survival outcomes (overall survival and post-progression survival) were evaluated using the Kaplan-Meier Plotter online tool (KMPlotter; http://kmplot.com/analysis/) (Lanczky and Gyorffy, 2021; Gyorffy, 2023). Analyses were restricted to the ovarian cancer dataset and limited to tumors of serous histology. Probes corresponding to *RBX1* (Affymetrix ID: 218117_at) were selected. Survival curves were generated in KMPlotter by dichotomizing patients at the median expression value. Hazard ratios (HRs) with 95% confidence intervals and p-values for logrank tests were calculated automatically. A p-value < 0.05 was considered statistically significant. Kaplan-Meier plots were exported as PDF files, and figures were prepared in Adobe Photoshop 2025.

### 2.2 Identification of candidate human *RBX1* SL interactors from model organisms

To identify candidate SL interactors of *RBX1*, a bioinformatic approach was employed in which SynLethDB (http://synlethdb.sist.shanghaitech.edu.cn/) (Guo et al., 2016) was queried for established *RBX1* interactors in model systems/organisms (e.g., *HRT1* in *Saccharomyces cerevisiae*). Briefly, SynLethDB (Guo et al., 2016) contains ∼50,000 SL interactors identified through genetic and biochemical assays, *in silico* analyses and text mining within various model organisms and human cell lines. Candidate SL interactors were exported into Excel (Microsoft).

### 2.3 Cell lines and culture

Heterozygous *RBX1* knockout (*RBX1* ^+/−^1; *RBX1* ^+/−^2) clones and non-targeting, sgRNA control clone (NT-Control) were previously generated in FT246 cells using a two-step CRISPR/Cas9 approach (Bungsy et al., 2021; Lepage et al., 2021). FT246 is a female non-malignant, non-transformed fallopian tube secretory epithelial cell line, which is a cellular precursor of HGSC (Karst et al., 2011; Perets et al., 2013; Labidi-Galy et al., 2017). FT246 clones were grown in DMEM/Ham’s F12 1:1 (HyClone, Logan, UT) supplemented with 2% Ultroser G (Pall France). Malignant HGSC cell lines, COV362 and OVCAR-3 were purchased from the American Type Culture Collection (ATCC) and were grown in DMEM supplemented with 10% FBS and RPMI supplemented with 10% FBS, 1 mM sodium pyruvate and 1x insulin-transferrin-selenium, respectively. FT246 clones, COV362, and OVCAR-3 were authenticated based on viability, doubling times, cellular morphology, spectral karyotyping and Western blot analyses (including hypomorphic *RBX1* expression relative to NT-Control) (Bungsy et al., 2021; Lepage et al., 2021). All cells were grown in a humidified incubator at 37 °C supplied with 5% CO_2_.

### 2.4 *CDK2* silencing and immunoblotting

*CDK2* silencing was performed as detailed elsewhere (McManus et al., 2009). Briefly, four individual ON-TARGETplus siRNA duplexes (Dharmacon, USA) targeting *CDK2* (siCDK2-1, -2, -3 or -4) or a pool (siCDK2-Pool) comprised of equimolar amounts of each individual siRNA were used along with a non-targeting siRNA control (siControl). *PLK1* silencing (siPLK1) was included as positive transfection control as it is an essential gene that induces extensive cell killing irrespective of *RBX1* genotype. Gene silencing was assessed by semi-quantitative western blots 6 days post-transfection as described (Bungsy et al., 2021; Lepage et al., 2021) using anti-CDK2 antibody (Abcam ab32147; 1:2,000) and anti-Cyclophilin B (loading control; Abcam ab16045; 1:25,000), with slight modifications. Briefly, following CPTS (Copper [II] Phthalocyanine 3,4′,4″,4‴ TetraSulfonic Acid Tetrasodium Salt) staining and prior to antibody incubations, some blots were cut to include relevant lanes and/or regions related to the proteins to be detected (CDK2, ∼33.9 kDa; Cyclophilin B, ∼23.7 kDa). All blots were imaged using a MyECL Imager (Thermo Scientific, Mississauga, ON, Canada) or a ChemiDoc MP imaging system (Bio-Rad, Mississauga, ON, Canada). Semi-quantitative analyses determined the relative CDK2 abundance, where signal intensities were first normalized to the corresponding loading control and are presented relative to siControl (100% expression). The two siRNAs (siCDK2-3 and siCDK2-4) inducing the greatest silencing in the NT-Control clones were employed in all direct SL tests. All figures were assembled in Photoshop.

### 2.5 Direct SL tests and Quantitative Imaging Microscopy

Following *CDK2* silencing or inhibition, quantitative imaging microscopy (QuantIM) was employed as described (McManus et al., 2009) to determine the number of nuclei (cells) remaining, with each experiment performed a minimum of three times. Briefly, 1,000 cells (*RBX1* ^+/−^ or NT-Control clones) were seeded into each well of a 96-well plate, permitted to attach (24 h) and were either transfected (siRNAs) or treated (SNS-032; DMSO [dimethyl sulfoxide]) in sextuplet. Cells were permitted to grow for 144 h (6-day) at which point they were fixed (4% paraformaldehyde), counterstained (Hoechst 33342) and imaged (Cytation 3; BioTek, United States). Sixteen (4 × 4 matrix) non-overlapping images were acquired from each well and the total number of nuclei was determined as described (McManus et al., 2009; Thompson and McManus, 2015). To account for potential differences in growth rates between the NT-Control/siControl and *RBX1* ^+/−^ clones or *RBX1*-silenced conditions, nuclear counts from each well were normalized to the average number of nuclear counts from the control conditions (siControl or DMSO for the siRNA or drug treatments, respectively). All normalized count data were imported into Prism, where statistical analyses (e.g., multiple paired t-tests) were performed. Briefly, pairwise t-tests directly compare means or distributions between two groups or conditions, allowing for focused evaluation of specific hypotheses about group differences while controlling for noise or confounding factors. Pairwise tests factor out individual-level variation (by using within-subject differences), making the analysis more sensitive to true treatment effects. The main assumptions are that observations are paired (not independent), the differences are normally distributed, and each pair is matched within the study design. Accordingly, paired t-tests are used to rigorously evaluate differences between two related samples, yield more precise and interpretable results for questions pertaining to treatment effects. For the FT246 clones, all experiments were conducted 3 times (N = 3), each with six technical replicates (n = 6) with a minimum of 500 nuclei imaged per technical replicate. Graphs were generated in Prism and exported into Photoshop, where figures were assembled.

### 2.6 Dual siRNA SL tests

SL tests were performed in COV362 and OVCAR-3 cells in which *RBX1* was co-silenced with *CDK2* using our established protocol (Sajesh and McManus, 2015). Briefly, 2,000 cells were dispensed into each well of a 96-well optically clear plate and permitted to attach and grow for 24 h. Cells were transfected in sextuplet with siRNAs targeting either *RBX1* (siRBX1-Pool) or siControl along with *CDK2* (siCDK2-Pool), with siPLK1 included as a transfection control. Cells were permitted to grow for 5-days at which point they were fixed (4% paraformaldehyde), counterstained (Hoechst) and subjected to QuantIM as described above. Briefly, data were imported into Excel where nuclear counts were normalized to the corresponding negative control (siControl). Relative nuclear numbers (%) were imported into Prism where multiple paired t-tests were conducted with a two-stage step-up (Benjamini, Krieger, and Yekutieli) correction for multiple comparisons using a false discovery rate (FDQ; Q) = 5%. A q-value < 0.05 deemed statistically significant. Each experiment was conducted once (N = 1) with six technical replicates (n = 6) and a minimum of 500 nuclei imaged per technical replicate. Graphs were generated in Prism and exported into Photoshop, where figures were assembled.

### 2.7 Dose response curves

Dose response curves were generated as detailed (Sajesh et al., 2013a) with slight modifications. Briefly, a 5-fold dilution series of SNS-032 (Selleck Chemicals; 1 pM to 100 μM) was performed in a 96-well plate with each concentration assessed in sextuplet. Cells were fixed 4 days post-treatment, counterstained (Hoechst 33342) and imaged as above. Nuclear (cell) counts were imported into Prism where average nuclear counts were normalized to the corresponding average counts from the vehicle control (DMSO). Standard dose response curves were generated and the EC_50_ (effective concentration at which 50% of the cells remained) values were automatically determined in Prism. Dose response curves were conducted 3 times (N = 3), each with six technical replicates (n = 6). The concentration of SNS-032 (160 nM) inducing the greatest reduction in *RBX1* ^+/−^ cell numbers relative to NT-Control was employed in subsequent direct tests.

### 2.8 Direct chemogenomic SL tests

Chemogenetic SL tests were performed in FT246 clones (*“RBX1* ^+/−^1”; *RBX1* ^+/−^2; NT-Control) and in malignant COV362 and OVCAR-3 cells in which *RBX1* had been silenced. For the FT246 clones, 4,000 cells were seeded into 96-well optical plates, permitted to attach and treated in sextuplet 24 h post-seeding with DMSO (vehicle control) or SNS-032 (160 nM). Cells were permitted to grow for an additional 3 days, at which point they were fixed, counterstained (Hoechst) and subjected to QuantIM as detailed above. Experiments were conducted in sextuplet and repeated 3 times. For the COV362/OVCAR-3 conditions, 2,000 cells were dispensed into each well of a 96-well optically clear plate and permitted to attach and grow for 24 h. Cells were transfected in sextuplet with pooled siRNAs targeting either *RBX1* (siRBX1-Pool) or siControl and permitted to grow for 24 h, at which point they were treated with DMSO or SNS-032 (160 nM). Following a 3-day incubation period, cells were fixed, counterstained and subjected to QuantIM as above. Briefly, data were imported into Excel where nuclear counts were normalized to the corresponding negative control (siControl). Relative nuclear numbers (%) were imported into Prism where multiple paired t-tests were conducted with a two-stage step-up (Benjamini, Krieger, and Yekutieli) correction for multiple comparisons using a false discovery rate (FDQ; Q) = 5%. A q-value <0.05 deemed statistically significant. For the FT246 clones, all experiments were conducted 3 times (N = 3), each with six technical replicates (n = 6) with a minimum of 500 nuclei imaged per technical replicate, while the COV362 and OVCAR-3 experiments were conducted once (N = 1), with six technical replicates (n = 6) and a minimum of 500 nuclei imaged per technical replicate. Graphs were generated in Prism and exported into Photoshop, where figures were assembled.

### 2.9 Real-time cell analyses

Real-time cell analyses (RTCA; i.e., growth curves) were performed in quadruplicate using an RTCA-DP (Agilent Technologies) as described (Sajesh and McManus, 2015). Briefly, RTCA is a label-free system that employs electrical impedance (termed cell index), as a measure of proliferation. It distinguishes cell cycle arrests from cell cytotoxicity, as a plateau in cell index is indicative of a cell cycle arrest, whereas a decrease in cell index is indicative of cell cytotoxicity (Solly, 2004). Briefly, 12,000 cells were seeded into each well of an E-Plate and growth was monitored every 30 min at 37 °C. DMSO or SNS-032 (160 nM) were supplemented into the medium when cell indices attained ∼20%–25% of their untreated maximum values (∼24 h post-seeding) and monitored for 6 days. Each experiment was performed in quadruplicate (n = 4) and repeated 3 times (N = 3).

### 2.10 Indirect immunofluorescence microscopy

Indirect immunofluorescence QuantIM was performed as detailed elsewhere (Sajesh et al., 2013a). Briefly, cells were treated with SNS-032 or DMSO, and DNA DSBs were assessed using a γ-H2AX antibody (Abcam; ab26350; 1:200) and the total number of DSBs (γ-H2AX) foci (Rogakou et al., 1998; Guppy and McManus, 2017) were enumerated from ≥250 nuclei/condition and statistically compared to vehicle control. A similar approach was employed to assess apoptosis using a cleaved Caspase-3 antibody (Abcam; ab13747; 1:200); however, an integrated cleaved Caspase-3 signal intensity was calculated for each nucleus by normalizing the total cleaved Caspase-3 intensity to the corresponding DAPI signal intensity (McManus and Hendzel, 2003). Importantly, all exposure times were first optimized and maintained constant across all conditions so that qualitative and quantitative comparisons could be made. All data were imported into Prism where γ-H2AX foci cumulative distribution frequencies were compared (two-sample Kolmogorov-Smirnov (KS) tests), and cleaved Caspase 3 signal intensities were assessed (Welch’s t-test) with p-values < 0.05 are considered significant.

## 3 Results

### 3.1 *RBX1* copy number losses and reduced mRNA expression are prevalent in HGSC

**FIGURE 1:**
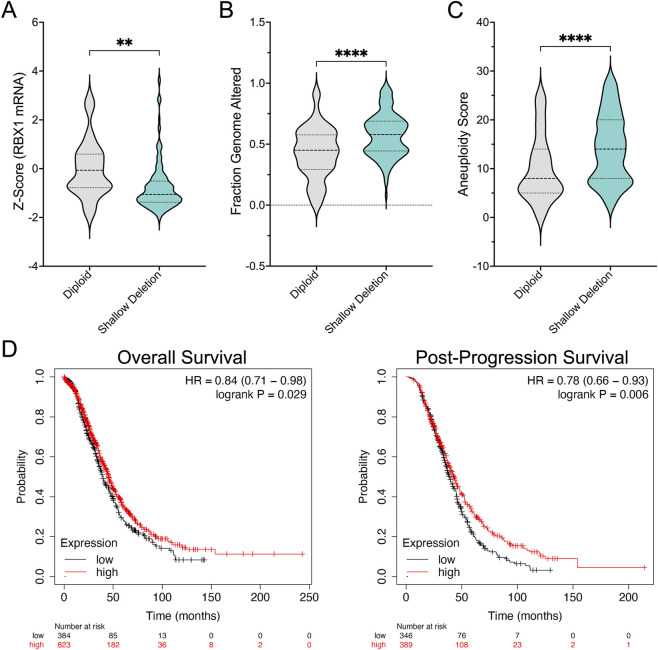
Clinical relevance of *RBX1* copy number losses and reduced expression in HGSC. **(A)** HGSC patient samples with shallow *RBX1* deletions (i.e., heterozygous loss; 166 cases) exhibit a significant decrease in *RBX1* mRNA expression levels relative to their diploid counterparts (23 cases; **; p-value < 0.01; two-tailed unpaired t-test). Horizontal lines identify interquartile range. **(B)** Cases with shallow *RBX1* deletions (322 cases) have significant increases in the fraction of the genome altered relative to diploid cases (51 cases; Mann-Whitney test; ****; p-value < 0.01). Horizontal lines identify interquartile range. **(C)** Cases with shallow *RBX1* deletions (318 cases) exhibit significant increases in aneuploidy scores relative to diploid cases (49 cases; Mann-Whitney tests). Horizontal lines identify interquartile range. **(D)** KM curves reveal significantly worse overall survival (left) and post-progression survival (right) for patients with reduced *RBX1* mRNA expression relative to those with high expression. Hazard ratios (HR) and logrank test p-values are indicated.

To explore the clinical impact *RBX1* copy number loss has in HGSC, bioinformatic analyses were performed on publicly available TCGA patient datasets available through cBioPortal (Cerami et al., 2012a; Gao et al., 2013; Hoadley et al., 2018). In keeping with its essential gene status (Meyers et al., 2017), deep deletions (i.e., homozygous loss) are rare, occurring in ∼1% (4/398) of HGSC cases, while shallow deletions (i.e., heterozygous loss) are present in ∼81% (322/398) of cases (Cerami et al., 2012a; Gao et al., 2013; Hoadley et al., 2018). Importantly, shallow deletions correspond with a significant reduction in *RBX1* expression at the mRNA level (Figure 1A); unfortunately, RBX1 protein abundance is not available. Shallow deletions are also associated with increases in genome instability within those patients, as evidenced by elevated fractions of the genome altered (Figure 1B) and higher aneuploidy scores (Figure 1C) (Cerami et al., 2012; Gao et al., 2013; Hoadley et al., 2018). These findings agree with our previous functional genetics study demonstrating that reduced *RBX1* expression induces CIN and promotes cellular transformation (i.e., early disease development) in HGSC contexts (Bungsy et al., 2021). Finally, reduced *RBX1* expression also corresponds with worse clinical outcomes (Figure 1D), as HGSC patients with low *RBX1* mRNA expression exhibit significantly worse overall and post-progression survival compared to those with high expression (Lanczky and Gyorffy, 2021; Gyorffy, 2023). Collectively, these findings support the hypothesis that reduced *RBX1* expression is a pathogenetic event in HGSC, rendering it an ideal candidate to target using a SL paradigm.

### 3.2 Cross-species approaches identify candidate SL interactors of *RBX1* and prioritize *CDK2*

Reasoning that strong candidate SL will be evolutionarily conserved across species, we sought to identify and prioritize a candidate *RBX1* SL interactor to pursue in subsequent direct SL tests. To this end, bioinformatic approaches were used to mine the SynLethDB database (Guo et al., 2016), an online repository containing SL datasets from model organisms and human cell lines. Briefly, 54 candidates were identified with established roles in DNA replication, DNA repair, and cell-cycle regulation (Supplementary Table S1), which included *CDK2* (Zimmermann et al., 2017). *CDK2* was prioritized for subsequent study for numerous reasons: 1) Cyclin E1 (*CCNE1*) normally binds CDK2 to activate it and drive S-phase entry and cell cycle progression (Honda et al., 2005); 2) *CCNE1* is an oncogene that is amplified in ∼20% of HGSCs (Hoadley et al., 2018) and overexpression induces CIN and promotes cellular transformation (Spruck et al., 1999; Karst et al., 2014); 3) *CCNE1* amplification correlates with increased *CDK2* expression and activity in HGSC (Etemadmoghadam et al., 2013); 4) Cyclin E1 is a target of the SCF complex that we previously determined increases in abundance following *RBX1* silencing or heterozygous loss (Bungsy et al., 2021); and, 5) HGSC cells with *CCNE1* amplification are sensitive to *CDK2* knockdown suggesting that these cells are dependent on CDK2 for their survival (Yang et al., 2015; Au-Yeung et al., 2017; Brown et al., 2022). Accordingly, we hypothesized that *CDK2* silencing or inhibition will subvert the oncogenic impact associated with increased Cyclin E1 abundance and induce SL killing in *RBX1*-deficient cells.

### 3.3 *CDK2* silencing induces decreases in *RBX1* ^+/−^ clones and *RBX1* silenced cell numbers

**FIGURE 2:**
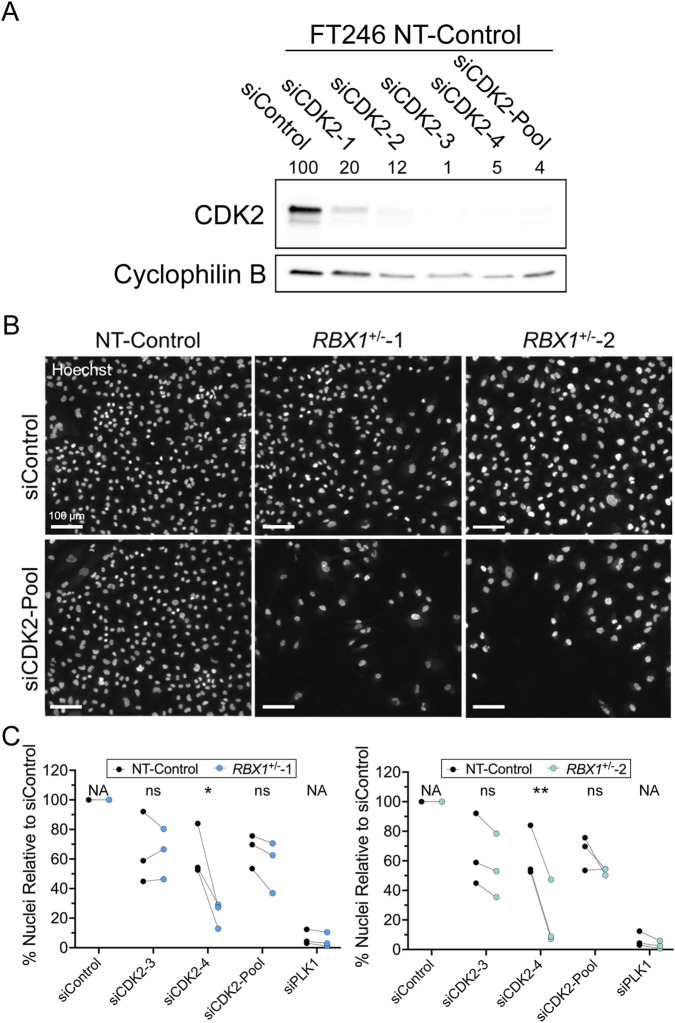
*CDK2* is a putative SL interactor of *RBX1*. **(A)** Western blot presenting CDK2 abundance in NT-Control FT246 cells following silencing with individual (siCDK2-1 to -4) or pooled (siCDK2-Pool) siRNAs relative to siControl, with Cyclophilin B serving as a loading control. CDK2 abundance was normalized to the respective loading control and is presented relative to the siControl (100%). Original unprocessed Western blot images are provided in Supplementary Figure S4A of Supplementary Material. **(B)** Representative low-resolution images depicting reduced nuclear (cell) numbers following *CDK2* silencing in both *RBX1* ^+/−^ clones relative to NT-Control. **(C)** Dot plots presenting the mean number of cells relative to siControl following *CDK2* silencing in NT-Control and *RBX1* ^+/−^ clones (biological replicate, N = 3; technical replicate, n = 6; >500 nuclei/technical replicate; ns = not significant; *, p-value < 0.05; **, p-value < 0.01).

Prior to assessing a putative SL interaction between *RBX1* and *CDK2*, we first confirmed both *RBX1* ^+/−^ clones exhibited increases in Cyclin E1 protein abundance (Supplementary Figure S1). It was also necessary to establish the silencing efficiency of four distinct siRNA duplexes targeting CDK2 (siCDK2-1 to -4); our previous study determined that the *RBX1* ^+/−^ clones reduced RBX1 levels to 78% (*RBX1* ^+/−^1) and 58% (*RBX1* ^+/−^2) of the NT-Control (Bungsy et al., 2021). As shown in Figure 2A, siCDK2-3 and siCDK2-4 are the two most efficient duplexes, which together with siCDK2-Pool were employed in all subsequent tests. To determine whether the *RBX1*:*CDK2* SL interaction is conserved in an HGSC context, we employed our established SL protocol (McManus et al., 2009; Sajesh and McManus, 2015; Jeusset and McManus, 2021). Briefly, *RBX1* ^+/−^1, *RBX1* ^+/−^2, and NT-Control clones were transfected with siRNAs (including siControl) and cells were permitted to grow for 6-days, at which point cells were fixed, counterstained, and subjected to QuantIM.

Overall, there were visual decreases in the number of *RBX1* ^+/−^1 and *RBX1* ^+/−^2 clones following silencing that were most pronounced within the siCDK2-4 and siCDK2-Pool conditions (Figure 2B). QuantIM identified a significant decrease (p-value < 0.05) in the number of *RBX1* ^+/−^1 cells following CDK2-4 silencing (Figure 2C; Supplementary Table S2) and trending decreases following siCDK2-Pool silencing (p-value > 0.05). Similarly, the *RBX1* ^+/−^2 clone exhibited a significant reduction (p-value < 0.01) in cell numbers following siCDK2-4 silencing and trending decreases following siCDK2-3 and siCDK2-Pool silencing (p-values > 0.05). It should be noted that unlike traditional SL studies that employ homozygous knockout models, our study employs clinically relevant, heterozygous models, and thus variation in the strength of the SL interaction is expected. Consistent with this notion, the SL impacts are most pronounced within the *RBX1* ^+/−^2 clone, which has the lowest abundance of RBX1 (45%) relative to the *RBX1* ^+/−^1 clone (68%) (Supplementary Figure S1). In agreement with a conserved SL interaction, *CDK2* silencing in two malignant HGSC cell lines, namely, COV362 and OVCAR-3 also induced a significant decreases in the number of *RBX1* silenced cells relative to siControl (Supplementary Figure S2; Supplementary Tables S3, S4). Collectively, these findings are consistent with *RBX1* and *CDK2* being SL interactors and identify CDK2 as a novel candidate drug target warranting further study.

### 3.4 *RBX1* ^+/−^ clones and *RBX1* silenced cells are hypersensitive to CDK2 inhibition with SNS-032

**FIGURE 3:**
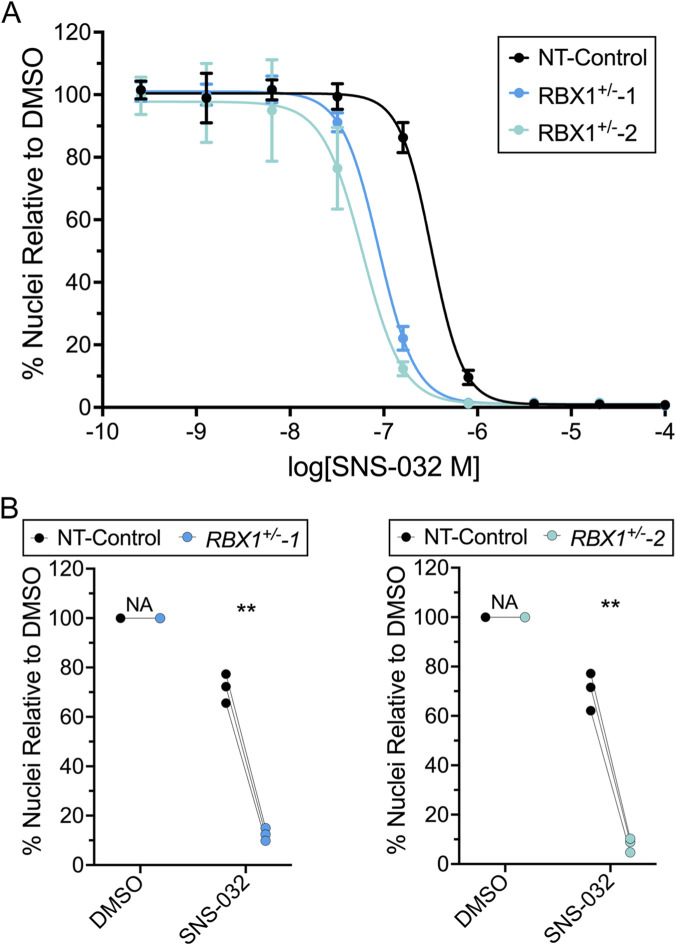
*RBX1* ^+/−^ clones are hypersensitive to SNS-032 treatments. **(A)** Dose response curves reveal *RBX1* ^+/−^ clones are hypersensitive to SNS-032 treatments relative to NT-Control. Data are normalized to the vehicle control (DMSO) and mean values ± standard deviation are presented (N = 2, n = 6; >500 nuclei/technical replicate). **(B)** Dot plots presenting the mean number of nuclei (cells) relative to DMSO following SNS-032 (160 nM) treatments. Paired t-tests identify significant decreases in cell numbers in both *RBX1* ^+/−^ clones relative to NT-Control (N = 3; n = 6; >500 nuclei/technical replicate; ns = not significant; **, p-value < 0.01).

To begin to assess the clinical potential of CDK2 as a novel drug target, we next determined whether SNS-032, a selective CDK2 inhibitor, could substitute for *CDK2* silencing and induce preferential killing within the *RBX1* ^+/−^ clones. However, it was first necessary to generate dose response curves to identify an optimal concentration of SNS-032 to employ in downstream direct tests. Accordingly, a 5-fold serial dilution curve was generated that revealed both *RBX1* ^+/−^ clones are hypersensitive to SNS-032 relative to NT-Control (Figure 3A), as the EC_50_ values calculated are 3.7- to 5.4-fold lower (*RBX1* ^+/−^1, 87.7 nM; *RBX1* ^+/−^2, 59.4 nM) than NT-Control (321 nM). Based on these curves, 160 nM of SNS-032 was identified as the optimal concentration for subsequent tests, as it induced a large decrease in number of *RBX1* ^+/−^ cells remaining, while having minimal impact on NT-Control.

Next, chemogenetic tests revealed that SNS-032 treatments induced significant decreases in the number of *RBX1* ^+/−^ cells remaining relative to NT-Control (Figure 3B; Supplementary Table S5). It should also be noted that the chemogenetic interactions are more pronounced than the original siRNA interactions identified above (see Figure 2C). More specifically, SNS-032 reduced *RBX1* ^+/−^1 and *RBX1* ^+/−^2 cell numbers to ∼12% and ∼8%, respectively, of the NT-Control (Figure 3B). Similarly, SNS-032 treatment corresponded with significant decreases in the number of *RBX1* silenced COV362 and OVCAR-3 cells relative to siControl (Supplementary Figure S3; Supplementary Tables S6, S7). Collectively, these findings identify a novel chemogenetic interaction between *RBX1* and SNS-032 and provide further support that *CDK2* is a SL interactor and drug target in multiple CRISPR/Cas9 and siRNA cellular contexts.

### 3.5 SNS-032 induces cell cytotoxicity in *RBX1* ^+/−^ cells

**FIGURE 4:**
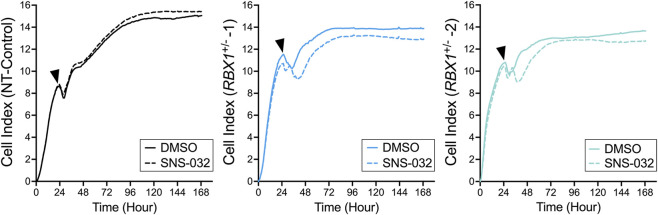
SNS-032 induces cytotoxicity in *RBX1* ^+/−^ clones. Representative real-time cell analysis curves for NT-control (left) and *RBX1* ^+/−^1 (middle) and *RBX1* ^+/−^2 (right) clones treated with DMSO (solid lines) or SNS-032 (160 nM; dashed lines). Cells were treated with DMSO or SNS-032 24 h post-seeding (arrowheads) and selective decreases in cell indices of *RBX1* ^+/−^ clones are evident at ∼48 h relative to NT-Control clones (N = 3; n = 4).

While the above findings support a potential SL interaction between *RBX1* and *CDK2*, they do not distinguish between cell cytotoxicity (i.e., induced cell killing) and cell cycle arrest. To distinguish these possibilities, RTCA was performed using our established approach (Sajesh and McManus, 2015; Guppy and McManus, 2017) in which all clones were treated with SNS-032 and compared to vehicle control (DMSO). As expected, SNS-032 treatments corresponded with decreases in cell indices (i.e., enhanced cytotoxicity) within the *RBX1* ^+/−^ clones relative to DMSO treated controls. More specifically, a rapid and greater decline (i.e., separation of curves) in the cell indices occurred for both *RBX1* ^+/−^ clones following SNS-032 treatment that did not occur within the NT-Control (Figure 4). Collectively, these data indicate that SNS-032 treatments preferentially induce cell cytotoxicity rather than a cell cycle arrest within the *RBX1* ^+/−^ clones and is consistent with SNS-032 inducing SL killing.

### 3.6 SNS-032 induces increases in DNA DSBs and apoptosis in *RBX1* ^+/−^ cells

**FIGURE 5:**
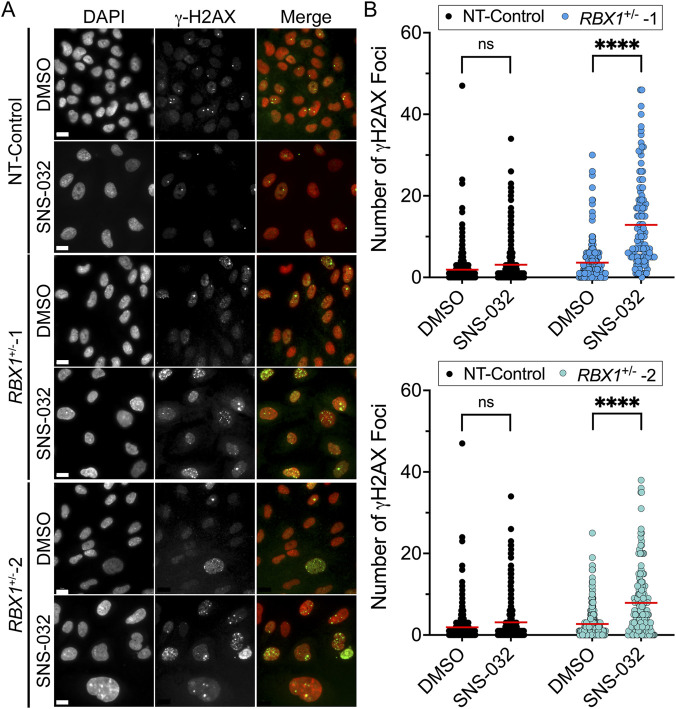
SNS-032 treatments induce increases in DNA DSBs within the *RBX1* ^+/−^ clones. **(A)** Representative low-resolution (10×) images of γ-H2AX abundance within NT-Control (top), *RBX1* ^+/−^1 (middle) and *RBX1* ^+/−^2 (bottom) clones treated with DMSO or SNS-032 (160 nM) for 48 h. Scale bars = 15 μm. **(B)** Dot plots comparing the number of γ-H2AX foci/cell following treatment with DMSO or SNS-032 in NT-Control and *RBX1* ^+/−^1 (top) or *RBX1* ^+/−^2 (bottom) clones. Red bars indicate mean number of foci/condition (N = 1; n ≥ 250 nuclei/condition; two-sample KS tests; ns = not significant; ****, p-value < 0.0001).

Having established that SNS-032 treatments induce cell cytotoxicity in *RBX1* ^+/−^ clones, we next sought to determine the underlying mechanism accounting for the SL phenotype. As SNS-032 treatments induce DNA DSBs in cervical cancer (Kang et al., 2018), we reasoned that a similar mechanism may contribute to the enhanced cell cytotoxicity observed within the *RBX1* ^+/−^ clones. Accordingly, an indirect immunofluorescent QuantIM approach (McManus and Hendzel, 2005; Guppy and McManus, 2017) was employed to statistically compare the number of γ-H2AX foci, a surrogate marker of DNA DSBs (Rogakou et al., 1998), present in *RBX1* ^+/−^ and NT-Control clones following SNS-032 treatments relative to vehicle controls (Figure 5). While increases in γ-H2AX foci were noted in all SNS-032 treated conditions, they were only deemed significant (p-value < 0.0001) within the *RBX1* ^+/−^ clones (Figure 5B; Supplementary Table S8). More specifically, SNS-032 treatments induced a 3.6- and 3.1-fold increase in the number of γ-H2AX foci within the *RBX1* ^+/−^1 and *RBX1* ^+/−^2 clones, respectively, while it corresponded with a non-significant, 1.3-fold increase within the NT-Control. Consistent with its established roles in maintaining genome stability by participating in DNA repair and replication (Jia et al., 2011; Xie et al., 2020), heterozygous loss of *RBX1* is associated with higher basal levels of γ-H2AX foci (DNA DSBs) relative to NT-Control (Figure 5B). For example, the mean number of γ-H2AX foci/cell is 64% and 22% greater in the DMSO-treated *RBX1* ^+/−^1 and *RBX1* ^+/−^2 clones, respectively, than the DMSO-treated NT-Control (Supplementary Table S8). Taken together, these data show that *RBX1* ^+/−^ cells have higher basal levels of γ-H2AX foci that become enhanced following SNS-032 treatments leading to significant increases in DNA DSBs.

### 3.7 SNS-032 treatments correspond with increases in a marker of apoptosis

**FIGURE 6:**
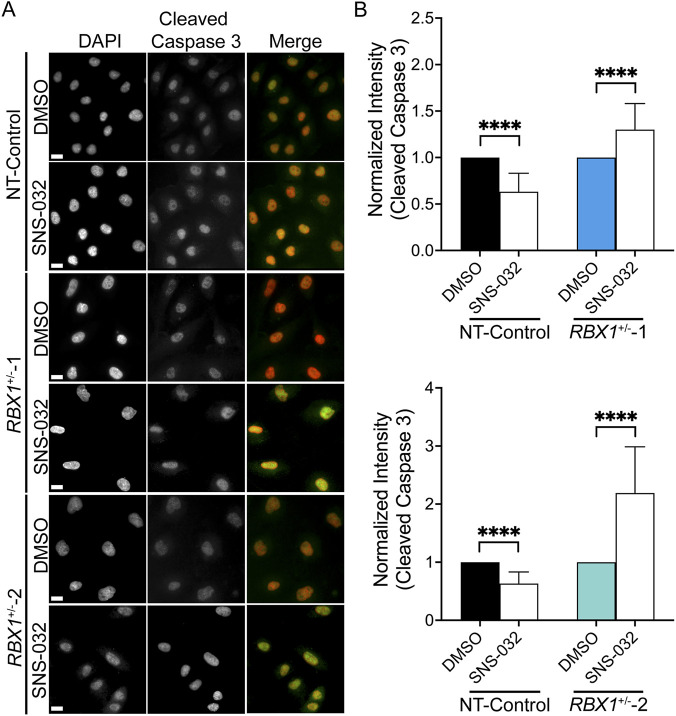
SNS-032 treatments correspond with increases in apoptosis in the *RBX1* ^+/−^ clones. **(A)** Representative low-resolution (10×) images of cleaved Caspase 3 abundance within NT-Control (top), *RBX1* ^+/−^1 (middle) and *RBX1* ^+/−^2 (bottom) clones treated with DMSO or SNS-032 (160 nM) for 48 h. Scale bars = 15 μm. **(B)** Bar graphs presenting the mean normalized cleaved Caspase 3 signal intensities ± standard deviation following treatments with DMSO or SNS-032 in NT-Control and *RBX1* ^+/−^1 (top) or *RBX1* ^+/−^2 (bottom) clones (N = 1; n ≥ 300 nuclei/condition; Welch’s t-tests; ns = not significant; ****, p-value < 0.0001).

Given that SNS-032 induces increases in DSBs and has previously been shown to induce apoptotic death in *CCNE1* amplified cancers (Chen et al., 2009; Yang et al., 2015), we next sought to determine whether apoptosis contributed to the SL killing in *RBX1* ^+/−^ cells. To investigate this possibility, we employed a similar indirect immunofluorescent QuantIM approach to that detailed above, focusing this time on the abundance of cleaved Caspase 3. Cleaved Caspase 3 is a well-established executioner caspase that operates at the intersection of the extrinsic and intrinsic pathways, and its increased abundance is a strong indicator of apoptotic induction (Salvesen, 2002; Fischer et al., 2003; Wanner et al., 2020). Consistent with SNS-032 inducing apoptosis, statistically significant increases in cleaved Caspase 3 signal intensities occurred within the *RBX1* ^+/−^ clones but not the NT-Control clone (Figure 6A; Supplementary Table S9). More specifically, SNS-032 treatments induced a 1.4-fold increase in cleaved Caspase 3 signal intensities within *RBX1* ^+/−^1 and a 2.3-fold increase in *RBX1* ^+/−^2, compared to their respective DMSO treated controls (Figure 6B). These findings agree with previous findings (Chen et al., 2009; Yang et al., 2015) and are consistent with SNS-032 inducing apoptosis and contributing to the SL killing observed in *RBX1* ^+/−^ cells.

## 4 Discussion

In this study, we employed bioinformatic, genetic, and functional assays to identify and validate *CDK2* as a novel SL interactor of *RBX1* in clinically relevant human HGSC contexts. To our knowledge, this is the first example of a SL interaction described in a heterozygous (e.g., *RBX1* ^+/−^) background rather than a homozygous (e.g., *RBX1* ^−/−^) knockout, highlighting the therapeutic potential of exploiting partial gene loss in cancer. Analysis of HGSC patient datasets revealed that heterozygous loss of *RBX1* occurs in the majority of HGSC cases that correlates with reduced transcript levels, increases in features associated with CIN, and worse clinical outcomes. Combined with our previous findings that reduced *RBX1* expression drives CIN and cellular transformation (Bungsy et al., 2021), these data underscore *RBX1* as a rationally selected candidate for SL targeting.

Our functional studies demonstrated that *CDK2* silencing preferentially reduced cell numbers of two *RBX1* ^+/−^ FT clones and in the *RBX1*-silenced COV362 and OVCAR-3 cell lines, and that treatment with SNS-032 reproduced this phenotype and corresponded with increases in DNA damage and apoptosis. While our initial emphasis was on the SCF complex, which regulates Cyclin E1 turnover and thereby CDK2 activity, we recognize that RBX1 is a core subunit of many Cullin-RING E3 ligases (CRLs). CRLs control proteasomal degradation of a vast array of substrates, including key regulators of the cell cycle, replication licensing, and DNA repair (Lydeard et al., 2013; Zhao and Sun, 2013). Many CDK2 targets or their binding partners are themselves regulated by CRLs, suggesting that the SL interaction may not result from disruption of a single pathway but rather reflect the combined destabilization of proteostasis (RBX1) and phosphorylation (CDK2) networks that normally safeguard genome stability. We note that, in the current study, the mechanistic link between *RBX1* loss, CDK2 dependency, and the observed increases in DNA damage and apoptosis is inferred from these functional phenotypes and from the known roles of RBX1-containing CRLs and CDK2, rather than delineated through direct pathway dissection. In keeping with our primary goal of establishing *RBX1* deficiency as a therapeutically tractable context for CDK2 inhibition in HGSC, we therefore focused on robust phenotypic readouts that are immediately actionable. A more detailed mapping of the upstream signaling and DNA damage response pathways that connect *RBX1* loss to CDK2 dependency will require dedicated mechanistic studies and represents an important direction for future work beyond the scope of the present study.

*CDK2* was identified as a putative SL interactor of *RBX1* through cross-species approaches (Guo et al., 2016; Zimmermann et al., 2017) and prioritized due to its clinical relevance in *CCNE1* amplified cancers (Yang et al., 2015; Au-Yeung et al., 2017; Brown et al., 2022; House et al., 2025), which RBX1-deficient cells phenocopy via Cyclin E1 accumulation (Bungsy et al., 2021; Supplementary Figure S1). While Cyclin E1-CDK2 drives G1/S transition, RB phosphorylation, and replication initiation, CDK2 also complexes with Cyclin A to promote S-phase progression and Cyclin Y, which has less well characterized functions (Wood and Endicott, 2018; Pellarin et al., 2025). Given RBX1-containing CRLs regulate multiple cyclins/cell cycle regulators, the SL interaction likely reflects broad CDK2 dysregulation across multiple phases rather than Cyclin E1 alone (Fagundes and Teixeira, 2021; Pellarin et al., 2025).

The selective CDK2 inhibitor, SNS-032, was purposefully selected as it is expected to exert anti-tumor effects by inducing apoptosis (Rees et al., 2022). In agreement with this, we show that SNS-032 treatments dramatically reduced the number of *RBX1* ^+/−^ FT and *RBX1*-silenced COV362 and OVCAR-3 cells, which is accompanied by increases in DNA DSBs and apoptosis. Our findings support those of Yang et al. (2015), who assessed the efficacy of SNS-032 in multiple ovarian cancer cell lines. Moreover, they determined that cells harboring *CCNE1* amplifications are ∼40-times more sensitive to SNS-032 than non-overexpressing cells and that this enhanced sensitivity corresponded with increased apoptotic death. Recently, new and more selective CDK2 inhibitors have been developed that have begun to enter clinical trials. For example, the VELA (Phase I/II) study is currently assessing the clinical utility of BLU-222, a potent and selective CDK2 inhibitor, for the treatment of advanced solid tumors, including HER2-negative breast cancers, ovarian cancers and cancers with genomic *CCNE1* amplification (Zarin et al., 2019; House et al., 2025). Although this work is ongoing, emerging data from preliminary *in vitro* studies show that ovarian cancer lines with *CCNE1* amplifications exhibit increased sensitivity relative to those with normal *CCNE1* expression. Collectively, these encouraging results together with those of the current study, support the need for additional pre-clinical studies to assess SNS-032 and emerging CDK2 inhibitors in contexts beyond those only having *CCNE1* amplifications and should be extended to include those harboring defects in *RBX1* and/or other SCF complex member genes. It is also worth noting that *CCNE1* amplifications only occur in ∼20% of HGSC cases (Bowtell et al., 2015), whereas ∼30–50% of all HGSCs exhibit increases in Cyclin E1 protein levels (Bowtell et al., 2015; Chan et al., 2020; Kang et al., 2023). Thus, additional mechanisms beyond genomic amplification, such as heterozygous loss of *RBX1* and/or reduced SCF complex function, are predicted to be sensitive to CDK2 inhibitors.

Although SNS-032 is regarded as a selective CDK2 inhibitor, it is important to note that it also inhibits additional CDKs, like CDK7 and CDK9, which play essential roles in transcriptional regulation and cellular stress responses (Sano et al., 2002; Chiang et al., 2024; Lee et al., 2024; Pellarin et al., 2025). Thus, we cannot fully exclude the possibility that inhibition of these additional kinases contributes to the SL phenotype observed in *RBX1*-deficient and silenced cells. Nevertheless, several lines of evidence support CDK2 as a primary mediator of this interaction: (1) siRNA-based silencing of *CDK2* in both *RBX1* ^+/−^ clones and two independent *RBX1*-silenced HGSC lines consistently reduced cell numbers; (2) *CDK2* was independently identified as an *RBX1* SL interactor in SynLethDB; and (3) an evolutionarily conserved SL relationship between *RBX1* and the *CDK2* ortholog *Cdc28* has been described in budding yeast (Zimmermann et al., 2017), consistent with prior studies demonstrating CDK2 dependency in *CCNE1* amplified ovarian and endometrial cancers (Yang et al., 2015; House et al., 2025). Accordingly, our data support *CDK2* as a biologically relevant SL interactor of *RBX1*, while not excluding a potential contributory role for *CDK7* and *CDK9*. A comprehensive dissection of the relative contributions of these additional CDKs, including the use of newer, highly selective CDK2 inhibitors, like BLU-222 or Tagtociclib (PF-07103091), and dedicated genetic and pharmacologic interrogation of CDK7 and CDK9, will be an important focus of future work but lies beyond the scope of the present study, which is centered on establishing the *RBX1*/*CDK2* SL axis in clinically relevant HGSC models.

Collectively, this work expands the scope of SL paradigms into heterozygous tumor suppressor states and identifies *RBX1* deficiency as a context in which CDK2 inhibition may be therapeutically leveraged. Although further mechanistic work is required to dissect the respective contributions of CDK2 and other CDKs, as well as to clarify how disruption of RBX1-containing CRL complexes intersects with CDK signaling pathways, our findings provide strong preclinical rationale for pursuing CDK2-targeted approaches in *RBX1*-deficient HGSC. Recent development of highly selective CDK2 inhibitors (House et al., 2025), such as those now entering clinical trials, further suggests that the *RBX1*/*CDK2* SL axis warrants continued investigation as it may offer a new precision medicine opportunity for HGSC patients.

## Data Availability

The original contributions presented in the study are included in the article/Supplementary Material, further inquiries can be directed to the corresponding author.
