# Supplementary material for: RBX1 loss sensitizes tubo-ovarian, high-grade serous ovarian cells to CDK2 inhibition by SNS-032
Source: Front Cell Dev Biol. 2026 Apr 28;14:1781550. doi: 10.3389/fcell.2026.1781550 (PMC13161083; doi:10.3389/fcell.2026.1781550)
Supplement: Supplementary file 1 [file DataSheet1.pdf]

Supplementary Material

1 Supplementary Tables

Supplementary Table 1. SynLethDB Identifies Putative SL Interactors of *RBX1*.

No.	SL Gene[A]	General Function/Pathway[B]	Model
1	*ARP2*	Actin polymerization and cytoskeleton dynamics	*Saccharomyces cerevisiae*
2	*CDC34*	Ubiquitin-proteasome pathway and cell cycle control	*Saccharomyces cerevisiae*
3	*CDC53*	SCF ubiquitin ligase complex and protein degradation	*Saccharomyces cerevisiae*
4	*GRR1*	SCF complex, ubiquitin-proteasome, cell cycle	*Saccharomyces cerevisiae*
5	*MAD1*	Spindle assembly checkpoint during mitosis	*Saccharomyces cerevisiae*
6	*MRE11*	DNA repair, recombination, genome stability	*Saccharomyces cerevisiae*
7	*SEC15*	Exocyst complex, vesicle trafficking, secretion	*Saccharomyces cerevisiae*
8	*SEC18*	SNARE protein, membrane fusion, vesicle trafficking	*Saccharomyces cerevisiae*
9	*SGS1*	DNA helicase, DNA repair, homologous recombination	*Saccharomyces cerevisiae*
10	*ATM*	DNA repair and cell cycle control	*Homo sapiens*
11	*BID*	Apoptosis (programmed cell death)	*Homo sapiens*
12	*BRCA1*	Tumor suppressor, DNA repair (homologous recombination)	*Homo sapiens*

13	*CDK2*	Cell cycle regulation by phosphorylating key proteins	*Homo sapiens*
14	*CDK4*	Cell cycle regulation by phosphorylating key proteins	*Homo sapiens*
15	*CDK6*	Cell cycle regulation by phosphorylating key proteins	*Homo sapiens*
16	*CETN2*	Centrosome duplication and separation	*Homo sapiens*
17	*DNTT*	V(D)J recombination during immune cell development	*Homo sapiens*
18	*EGFR*	Cell growth, proliferation, and differentiation	*Homo sapiens*
19	*ERCC1*	DNA repair, nucleotide excision repair	*Homo sapiens*
20	*GTF2H1*	Transcription initiation, DNA repair	*Homo sapiens*
21	*H2AC8*	Chromatin structure, gene regulation	*Homo sapiens*
22	*H4C1*	Chromatin structure, gene expression	*Homo sapiens*
23	*IGF1*	Cell growth, development, and physiological regulation	*Homo sapiens*
24	*IGFBP3*	Regulation of insulin-like growth factors	*Homo sapiens*
25	*IGHMBP2*	DNA repair, maintenance of genomic stability	*Homo sapiens*
26	*KDR*	Blood vessel formation	*Homo sapiens*
27	*MAPK12*	Intracellular signaling	*Homo sapiens*
28	*MDM2*	Negative regulator of p53 tumor suppressor	*Homo sapiens*
29	*MT1X*	Metal ion homeostasis, detoxification	*Homo sapiens*

30	*MTOR*	Regulation of cell growth and metabolism	*Homo sapiens*
31	*NHP2*	RNA processing, telomere maintenance	*Homo sapiens*
32	*PARP1*	DNA repair, regulation of chromatin structure	*Homo sapiens*
33	*PARP2*	DNA repair, especially in base excision repair	*Homo sapiens*
34	*PMS2*	DNA mismatch repair	*Homo sapiens*
35	*POLM*	Non-homologous end joining during DNA repair	*Homo sapiens*
36	*POLD1*	DNA replication and repair	*Homo sapiens*
37	*POLD4*	DNA replication and repair	*Homo sapiens*
38	*POLE*	DNA replication and repair	*Homo sapiens*
39	*POLM*	DNA repair	*Homo sapiens*
40	*POLR2A*	Transcription as part of RNA polymerase II complex	*Homo sapiens*
41	*RAD23B*	DNA repair and proteasomal degradation	*Homo sapiens*
42	*RAD51*	Homologous recombination, DNA repair	*Homo sapiens*
43	*RAD9A*	Component of RAD9-HUS1-RAD1 complex, DNA repair	*Homo sapiens*
44	*RELA*	Regulation of immune response, inflammation	*Homo sapiens*
45	*RPL13A*	Component of the ribosome, involved in protein synthesis	*Homo sapiens*
46	*RRM1*	Involved in DNA synthesis	*Homo sapiens*
47	*SESN2*	Regulation of cellular responses to stress, including DNA damage	*Homo sapiens*

48	*SSBP1*	DNA replication and repair	*Homo sapiens*
49	*TNFRSF10B*	Induces apoptosis	*Homo sapiens*
50	*UNG*	Base excision repair pathway	*Homo sapiens*
51	*USP1*	Deubiquitinates and stabilizes proteins in DNA repair	*Homo sapiens*
52	*WRAP53*	Telomere maintenance, DNA repair	*Homo sapiens*
53	*XRCC2*	Homologous recombination, DNA repair	*Homo sapiens*
54	*ZBTB32*	Transcriptional repressor with a role in immune response	*Homo sapiens*

[A]Published SL interactors of *RBX1* identified in SynLethDB (Guo et al., 2016).
[B]Functions/Pathways of Putative SL interactors of *RBX1* identified in GeneCards (Safran et al., 2021).

Supplementary Table 2. Direct SL Tests Identify *CDK2* as a Putative *RBX1* SL Interactor.

Cell Line	Condition	N[A]	p-value[B]	Significance[C]
NT-Control	siControl	3	NA	NA
	siCDK2-3		NA	NA
	siCDK2-4		NA	NA
	siCDK2-Pool		NA	NA
RBX1$^{+/-}$-1	siControl	3	NA	NA
	siCDK2-3		0.8938	ns
	siCDK2-4		0.0469	*
	siCDK2-Pool		0.1125	ns
RBX1$^{+/-}$-2	siControl	3	NA	NA
	siCDK2-3		0.0507	ns
	siCDK2-4		0.0047	**
	siCDK2-Pool		0.2263	ns

[A]6 Technical replicates (n) per biological replicate (N).
[B]p-values calculated from paired *t* tests comparing the mean number of NT-Control and *RBX1*$^{+/-}$ clones following *CDK2* silencing.
[C]Significance level (ns = not significant; *, p-value < 0.05; **, p-value < 0.01).

Supplementary Table 3: *RBX1* and *CDK2* are SL Interactors in COV362 Cells.

Condition	Mean (siControl)^{A}	Mean (siRBX1)^{A}	Diff^{B}	SE of Diff^{C}	t ratio	Df^{D}	q-value^{E}
siControl	100.0	100.0	0.000	0.000			
siCDK2-Pool	79.41	62.26	17.16	5.219	3.288	5	0.022850
siPLK1-Pool	9.093	9.613	-0.5199	1.040	0.5000	5	0.335113

^{A}mean normalized number of nuclei (cells) relative to siControl
^{B}Difference (Diff); Mean (siControl) – Mean (siRBX1-Pool)
^{C}Standard error of the difference (SE of Diff)
^{D}Degrees of freedom (DF)
^{E}q-value; paired t-tests with two-stage step-up (Benjamini, Krieger, and Yekutieli) correction for multiple comparisons with a false discovery rate (FDR; Q) = 5%; a q-value <0.05 is considered significant

Supplementary Table 4: *RBX1* and *CDK2* are SL Interactors in OVCAR-3 Cells.

Condition	Mean (siControl)^{A}	Mean (siRBX1)^{A}	Diff^{B}	SE of Diff^{C}	t ratio	Df^{D}	q-value^{E}
siControl	100.0	100.0	0.000	0.000			
siCDK2-Pool	76.58	50.45	26.13	4.077	6.409	5	0.001440
siPLK1-Pool	2.560	2.088	0.4716	0.564	0.836	5	0.231784

^{A}mean normalized number of nuclei (cells) relative to siControl
^{B}Difference (Diff); Mean (siControl) – Mean (siRBX1-Pool)
^{C}Standard error of the difference (SE of Diff)
^{D}Degrees of freedom (DF)
^{E}q-value; paired t-tests with two-stage step-up (Benjamini, Krieger, and Yekutieli) correction for multiple comparisons with a false discovery rate (FDR; Q) = 5%; a q-value <0.05 is considered significant

Supplementary Table 5. SNS-032 Treatments Corresponds with Reduced Cell Numbers in *RBX1*$^{+/-}$ Clones.

Cell Line	Condition	N[A]	Mean Frequency of Cells Remaining[B]	p-value[C]	Significance[D]
NT-Control	DMSO	3	100%	NA	NA
	SNS-032		100%	NA	NA
RBX1$^{+/-}$-1	DMSO	3	71.7%	NA	NA
	SNS-032		12.4%	0.0011	**
RBX1$^{+/-}$-2	DMSO	3	70.3%	NA	NA
	SNS-032		7.9%	0.0019	**

[A]6 Technical replicates (n) per biological replicate (N).
[B]Mean frequency of cells remaining relative to untreated control.
[C]p-values calculated from paired *t* tests comparing the mean number of NT-Control and *RBX1*$^{+/-}$ clones following SNS-032 treatment.
[D]Significance level (ns = not significant; **, p-value < 0.01).

Supplementary Table 6: SNS-032 Induces a Preferential Decrease in *RBX1* Silenced COV362 Cells.

Condition	Mean (siControl)[A]	Mean (siRBX1)[A]	Diff[B]	SE of Diff[C]	t ratio	Df[D]	q-value[E]
DMSO	100.0	100.0	0.000	0.000			
SNS-032	82.50	60.29	22.22	7.103	3.128	5	0.027306

[A]Mean normalized number of nuclei (cells) relative to siControl
[B]Difference (Diff); Mean (siControl) – Mean (siRBX1-Pool)
[C]Standard error of the difference (SE of Diff)
[D]Degrees of freedom (DF)
[E]q-value; paired t-tests with two-stage step-up (Benjamini, Krieger, and Yekutieli) correction for multiple comparisons with a false discovery rate (FDR; Q) = 5%; a q-value <0.05 is considered significant

Supplementary Table 7: SNS-032 Induces a Preferential Decrease in *RBX1* Silenced OVCAR-3 Cells.

Condition	Mean (siControl)[A]	Mean (siRBX1)[A]	Diff[B]	SE of Diff[C]	t ratio	Df[D]	q-value[E]
DMSO	100.0	100.0	0.000	0.000			
SNS-032	65.00	28.85	36.15	5.536	6.530	5	0.001323

[A]Mean normalized number of nuclei (cells) relative to siControl
[B]Difference (Diff); Mean (siControl) – Mean (siRBX1-Pool)
[C]Standard error of the difference (SE of Diff)
[D]Degrees of freedom (DF)
[E]q-value; paired t-tests with two-stage step-up (Benjamini, Krieger, and Yekutieli) correction for multiple comparisons with a false discovery rate (FDR; Q) = 5%; a q-value <0.05 is considered significant

Supplementary Table 8. SNS-032 Treatment Induces Significant Increases in γ-H2AX in *RBX1*$^{+/-}$ Clones

Cell Line	Condition	N[A]	Mean γ-H2AX Foci	Fold Increase[B]	p-value[C]	Significance[D]
NT-Control	DMSO	3	2.206		NA	NA
	SNS-032		2.913	1.3	0.1084	ns
RBX1$^{+/-}$-1	DMSO	3	3.609		NA	NA
	SNS-032		12.872	3.6	<0.0001	****
RBX1$^{+/-}$-2	DMSO	3	2.695		NA	NA
	SNS-032		8.295	3.1	<0.0001	****

[A]≥ 100 nuclei analyzed per condition (n) per biological replicate (N).
[B]Fold increase in mean number of γ-H2AX foci relative to the corresponding DMSO control.
[C]p-values calculated from two-sample KS tests comparing the distribution of γ-H2AX foci within NT-Control and *RBX1*$^{+/-}$ clones following SNS-032/DMSO treatment within a representative biological replicate.
[C]Significance level (ns = not significant; ****, p-value < 0.0001).

Supplementary Table 9. SNS-032 Treatments Correspond with Significant Increases in Cleaved Caspase 3 in *RBX1*$^{+/-}$ Clones.

Cell Line	Condition	N[A]	p-value[B]	Significance[C]
NT-Control	DMSO	1	NA	NA
	SNS-032		<0.0001	****
RBX1$^{+/-}$-1	DMSO	1	NA	NA
	SNS-032		<0.0001	****
RBX1$^{+/-}$-2	DMSO	1	NA	NA
	SNS-032		<0.0001	****

[A]≥ 100 nuclei analyzed per condition (n) per biological replicate (N).
[B]p-values calculated from Welch's *t*-tests comparing the DMSO-normalized total signal intensity of Cleaved Caspase 3 within NT-Control and *RBX1*$^{+/-}$ clones following SNS-032/DMSO treatment.
[C]Significance level (ns = not significant; ****, p-value < 0.0001).

2 Supplementary Figures

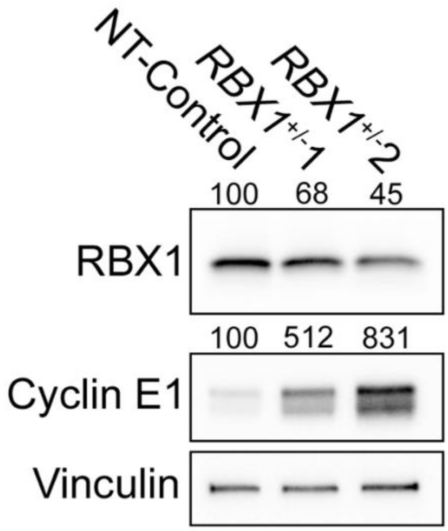

Supplementary Figure 1: Heterozygous Loss of *RBX1* Corresponds with Increases in Cyclin E1 Abundance. Western blot depicting reduced RBX1 abundance in *RBX1*$^{+/-}$1 and *RBX1*$^{+/-}$2 clones relative to NT-Control. RBX1 loss also corresponds with increases in Cyclin E1 abundance. Semi-quantitative analyses were conducted whereby RBX1 and Cyclin E1 abundance were normalized to their respective loading control and are presented relative to NT-Control (100%). Densitometry analyses for RBX1, Cyclin E1 and Vinculin were performed in Fiji and are indicate with the relative abundance (%) of RBX1 and Cyclin E1 presented above each lane. Original unprocessed western blot images are provided in Supplementary Figure 4B (below).

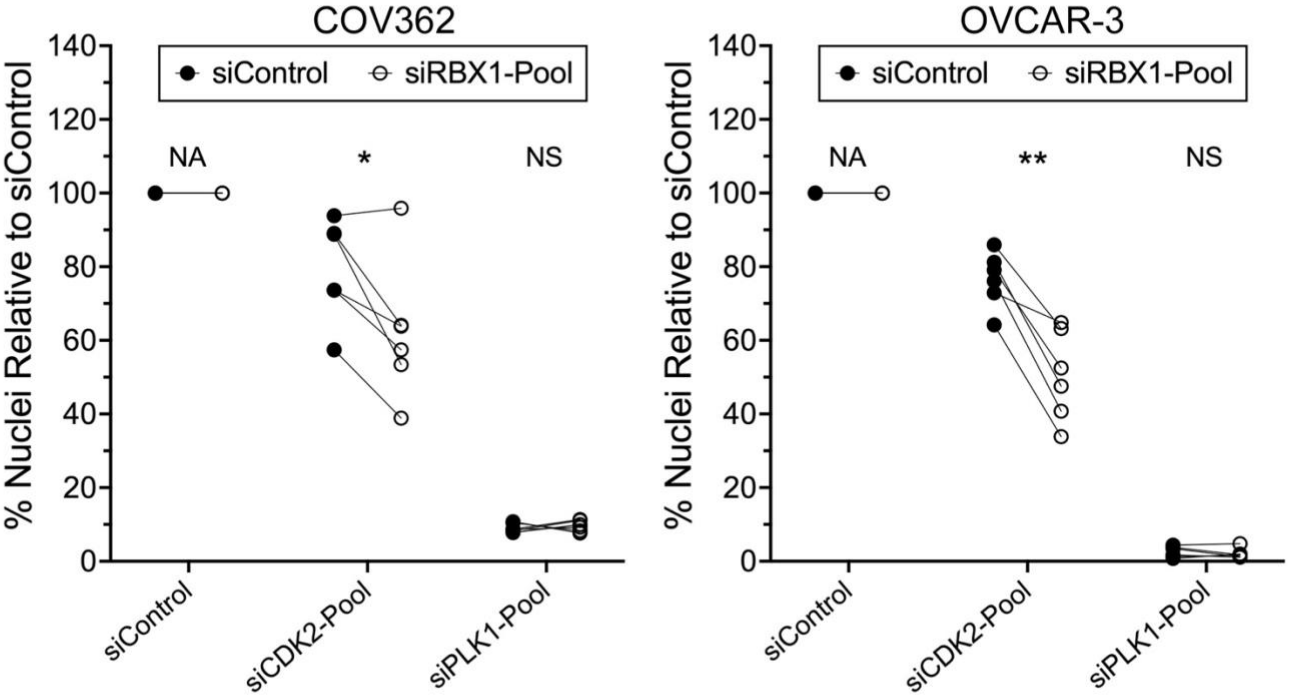

Supplementary Figure 2: *CDK2* Silencing Induces a Preferential Reduction in *RBX1* Silenced HGSC Cells. *CDK2* silencing induces a preferential reduction in the relative number of nuclei (cells) in *RBX1* silenced COV362 (left) and OVCAR-3 (right) cells relative to siControl. *PLK1* silencing serves as a transfection control. Each condition conducted in sextuplet and statistically assessed using multiple paired t-tests with an FDR = 0.05 using a two-stage step-up (Benjamini, Krieger, and Yekutieli) with a $q < 0.05$ considered statistically significant (**, q-value < 0.01; N = 1; n = 6; ≥ 250 cells/technical replicate).

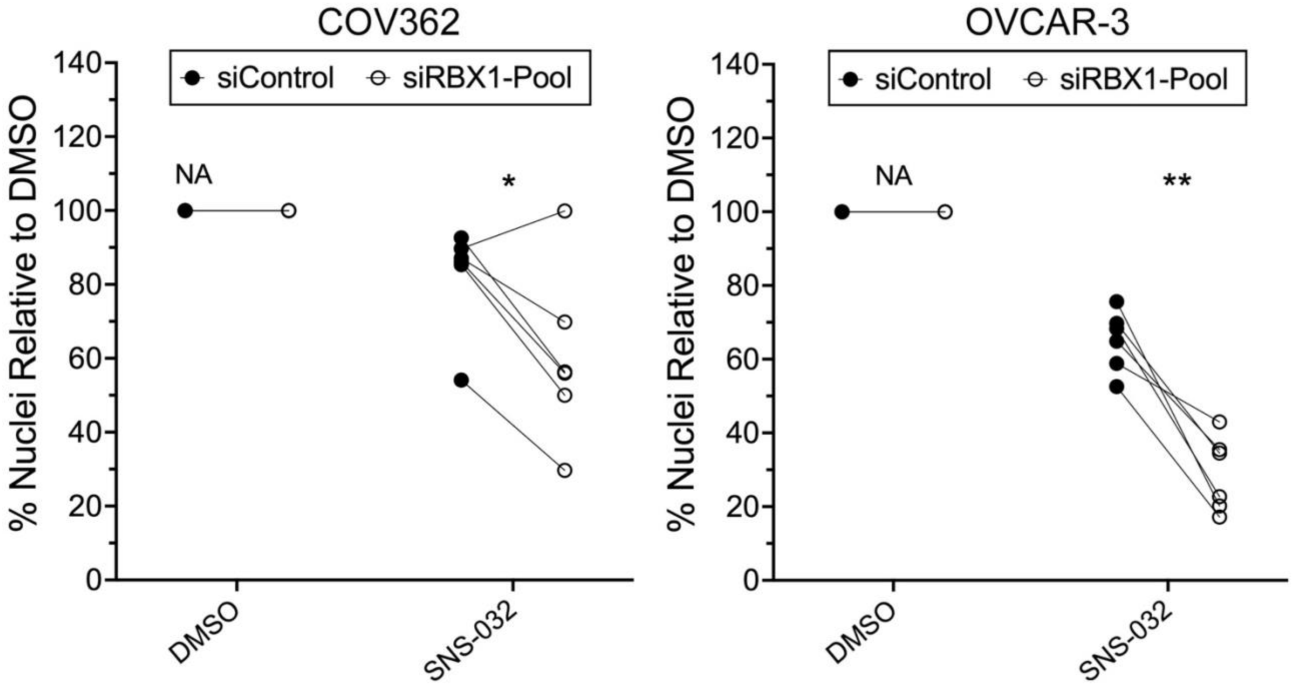

Supplementary Figure 3: SNS-032 Treatments Corresponds with a Reduction in *RBX1* Silenced HGSC Cells. Treatment of *RBX1* silenced COV362 (left) and OVCAR-3 (right) cells corresponded with a significant reduction in nuclear counts (cells) relative to similarly treated siControl cells. Each condition conducted in sextuplet and assessed using multiple paired t-tests with an FDR = 0.05 using a two-stage step-up (Benjamini, Krieger, and Yekutieli) with a $q < 0.05$ considered statistically significant (*, q-value < 0.05; **, q-value < 0.01; N = 1; n = 6; ≥ 250 cells/technical replicate).

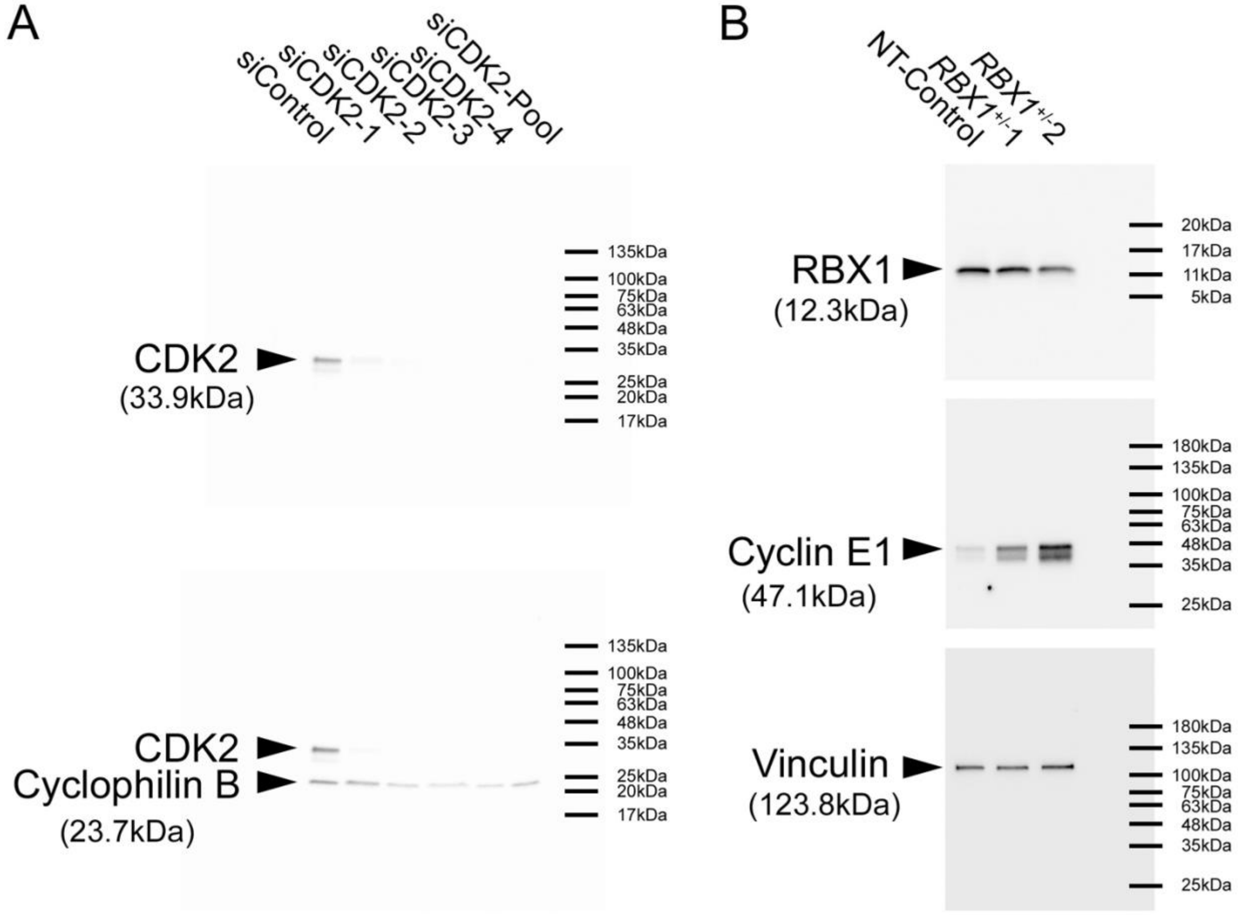

Supplementary Figure 4: Supporting Original Unprocessed Western Blots. (A) Original unprocessed western blot images corresponding to the optimized and cropped versions presented in Figure 2A. Conditions (lanes) are indicated at the top, while the specific antibody targets are presented to the left of each blot. The positions and sizes of the molecular weight markers (BLUelf pre-stained protein ladder; FroggaBio) are indicated by the lines to the right of each blot. **(B)** Original unprocessed western blot images corresponding to the optimized and cropped versions presented in Supplementary Figure 1. Specific clones assessed in the blots (lanes) are indicated at the top, while the specific antibody targets are presented to the left of each blot. The positions and sizes of the molecular weight markers (BLUelf pre-stained protein ladder; FroggaBio) are indicated by the lines to the right of each blot.
